# O_2_-Generated Electrical and Mechanical Properties of Polyphenol-Mediated Hydrogel Sensor

**DOI:** 10.3390/gels11080566

**Published:** 2025-07-22

**Authors:** Sunu Hangma Subba, A Hyeon Kim, Anneshwa Dey, Byung Chan Lee, Sung Young Park

**Affiliations:** 1 Department of IT and Energy Convergence, Korea National University of Transportation, Chungju 27469, Republic of Korea; sunuhangma23@gmail.com (S.H.S.); danwesha500@gmail.com (A.D.); 2 Department of Chemical and Biological Engineering, Korea National University of Transportation, Chungju 27469, Republic of Korea; dkgusaks@gmail.com; 3 Department of Environmental Engineering, Korea National University of Transportation, Chungju 27469, Republic of Korea

**Keywords:** oxygen-sensitive, hydrogel biosensor, cancer sensing, ROS scavenging

## Abstract

The tumor microenvironment contains distinctive biomarkers, including acidic pH, elevated levels of reactive oxygen species (ROS), and hypoxia, necessitating the development of efficient biosensors for simplified cancer detection. This study presents an O_2_-responsive hydrogel biosensor composed of [1,1′-biphenyl]-2,2′,4,4′,5,5′-hexaol (HDP) and polyvinyl alcohol (PVA) that exploits polyphenol-mediated interactions under N_2_ and O_2_ microenvironments. The oxidative susceptibility of the polyphenolic HDP moiety influences its distinct mechanical, physical, and electrochemical properties, allowing the differentiation between cancerous and normal cells. The in vitro assessments with cancer cell lines (HeLa and B16F10) and normal cell lines (CHO-K1) enabled distinctive electrical and mechanophysical outputs, as evidenced by enhanced mechanical compressive modulus and high conductivity, regulated by normoxic cellular states. In addition, the inherent ROS-scavenging capability of the HDP–PVA hydrogel sensor supports its potential application in hypoxia-related diseases, including cancer.

## 1. Introduction

Hypoxia, characterized by reduced oxygen levels, plays a critical role in the progression of various diseases, including cancer [1,2,3]. It significantly influences the tumor microenvironment and contributes to cancer development and metastasis [1,2,4]. As a result, several biomarkers such as acidic pH and elevated reactive oxygen species (ROS) levels have been used to detect cancer, including those targeting hypoxic conditions within tumors. Because oxygen availability governs hypoxic progression, technologies that use hypoxia as a diagnostic marker hold great promise for advancing cancer detection strategies. Therefore, the design of a responsive platform capable of altering its physical, mechanical, and electrical properties, including its ROS-scavenging ability, in response to hypoxic (low oxygen) vs. normoxic (normal oxygen) conditions presents a novel and practical approach for disease sensing, particularly for cancer.

In recent years, hydrogels have garnered significant attention for their application in various biomedical diagnostics, owing to their tunable mechanophysical characteristics under physiological markers such as glucose, enzymes, reactive oxygen species (ROS), good biocompatibility mimicking tissues, and ease of functionalization, making them ideal for biosensor development [5,6,7]. Recent studies have demonstrated the potential of oxygen-sensitive and redox-responsive hydrogels in biomedical diagnostics and therapy. For example, injectable hydrogels with ROS-scavenging and antioxidant ability have been developed to enhance cell viability and tissue regeneration, demonstrating the potential of redox-responsive systems for biomedical applications [8,9]. Additionally, polyphenol-based hydrogels, including iron chelators and lipoic acid, demonstrated antioxidant and anti-inflammatory capabilities [10]. Recent studies have also reported the use of oxygen-generating and oxygen-releasing hydrogels for tissue repair and bone regeneration, highlighting the clinical potential of oxygen-sensitive materials [11].

Hydrogels modified with stimuli-responsive functional materials can also be tailored to respond to specific microenvironmental cues such as temperature, pH, and oxygen, making them particularly useful for disease detection, including cancer diagnostics [12,13,14]. Moreover, integrating conductive polymers or nanomaterials into the hydrogel facilitates the fabrication of platforms that transduce biological events into electrical signals, enabling sensitive and real-time detection [15,16]. Several polyphenol-based hydrogels, such as tannic acid (TA)-based systems, primarily offer antioxidant or adhesive functionalities but lack dynamic responsiveness to oxygen levels [17,18]. However, most existing hydrogel biosensors rely on single-stimulus responsiveness and often lack specificity to complex pathological environments. Additionally, wireless integration of hydrogel biosensors for on-site diagnostics remains underexplored. To address these gaps, the HDP–PVA hydrogel biosensor presented in this study offers several key advantages over state-of-the-art oxygen-sensitive hydrogels. It combines multimodal responsiveness, including mechanical, electrochemical, and antioxidant (ROS-scavenging) properties, with robust chemical stability and biocompatibility, offering a potential for disease diagnosis, including cancer detection.

The objective of this study is to develop a hypoxia-responsive (HDP–PVA) hydrogel biosensor composed of polyvinyl alcohol (PVA) and polyphenolic [1,1′-biphenyl]-2,2′,4,4′,5,5′-hexaol (HDP), capable of sensing the cancer microenvironment through changes in mechanical, electrochemical, and ROS-scavenging properties. We hypothesize that the HDP–PVA hydrogel uses the oxidative susceptibility of the polyphenolic HDP component to form quinone derivatives under normoxic conditions, leading to distinct alterations in mechanical, physical, and electrical properties of the sensor. The cancer-sensing ability was validated by treating cancer cells (HeLa and B16F10 cells) and normal cells (CHO-K1) with the HDP–PVA hydrogel. Cancer cells, which are typically hypoxic, induced a markedly different response in the hydrogel compared to normal cells, as evidenced by their distinct mechanical and electrical profiles. Moreover, the sensor provides real-time wireless cancer-sensing ability when integrated with a smartphone using Bluetooth. These findings demonstrate the potential of the engineered HDP–PVA hydrogel biosensor as a simple and convenient cancer-sensing technology.

## 2. Results and Discussion

### 2.1. Fabrication and Characterization of the O_2_-Responsive HDP–PVA Hydrogel

**Figure 1:**
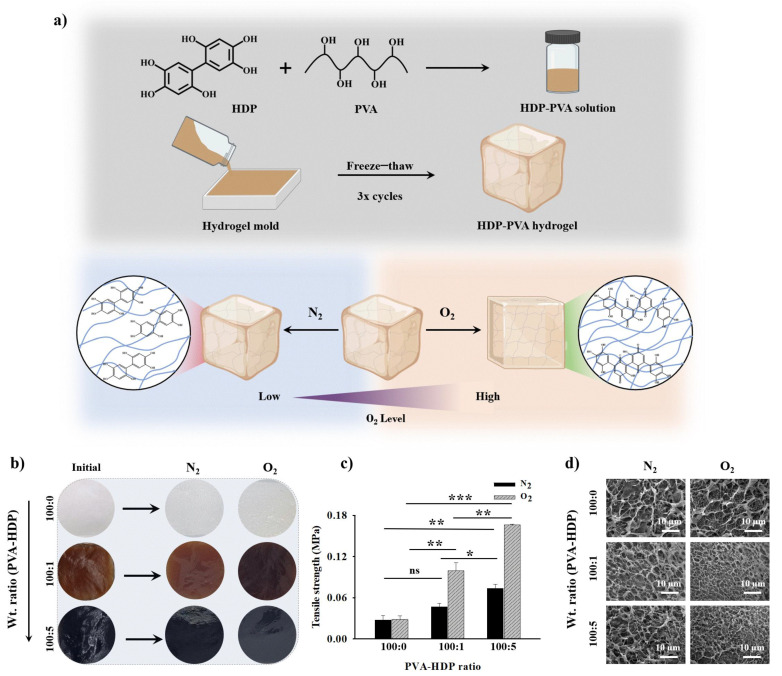
(**a**) Schematic representation of the fabrication of HDP–PVA hydrogel and its oxygen-specific mechanism. (**b**) Color change representation, (**c**) tensile strength, and (**d**) scanning electron microscopy (SEM) images of HDP–PVA hydrogel in N_2_ and O_2_ conditions. Statistical analysis performed using variance (ANOVA) followed by Tukey’s multiple comparison test (* *p* < 0.05, ** *p* < 0.01, and *** *p* < 0.001, and ns indicates non-significant differences for *n* = 3 samples).

An O_2_-responsive HDP–PVA hydrogel was designed for cancer detection by exploiting the oxidation susceptibility of the oxygen-sensitive polyphenolic HDP moiety, which modulated the mechanical and electrical properties of the hydrogel biosensor through oxygen-mediated interactions (Figure 1a). The selection of polyphenolic HDP is central to the functionality of the hydrogel. Structurally, HDP possesses six hydroxyl groups on a biphenyl core, giving it high polarity and strong hydrogen bonding potential. These features enhance the mechanical properties by promoting physical crosslinking through hydrogen bonding interactions with PVA chains. The hydroxyl groups also confer redox reactivity, allowing HDP to undergo oxidation under O_2_ conditions, altering the electronic and structural characteristics of the hydrogel. Furthermore, the polyphenolic structure of HDP imparts biochemical functionality by acting as a ROS scavenger, neutralizing oxidative stress often present in cancerous microenvironments [19]. Thus, HDP serves as a multifunctional component that imparts oxygen sensitivity, mechanical tunability, electrochemical responsiveness, and antioxidant behavior to the hydrogel matrix. PVA, a synthetic polymer, was selected for its excellent hydrophilicity, chemical stability, and biocompatibility, which makes it highly suitable for forming hydrogel matrices that are both mechanically robust and capable of incorporating functional additives [20,21,22]. The combination of HDP and PVA enables the fabrication of a cancer microenvironment-responsive sensing platform.

The redox-responsive characteristics of the HDP nanoparticles were investigated in N_2_ and O_2_ environments. As shown in Figure S1a, free HDP exposed to N_2_ exhibited a strong absorbance peak at 280 nm, corresponding to the π–π* transitions of catechol moieties [19,23]. In contrast, under aerobic conditions, a prominent absorbance peak emerged at approximately 410 nm, attributed to n–π* transitions characteristic of quinone formation, indicating the oxidative conversion of HDP in the presence of oxygen. Additional evidence of chemical stability of HDP in diverse environments was provided by previously reported ^1^H-NMR analysis, which demonstrated no significant chemical shift or variation in peak areas, thereby confirming the structural integrity of HDP [19]. Furthermore, cyclic voltammetry (CV) was employed to further corroborate the redox-sensitive behavior of polyphenolic HDP. Figure S1b illustrates a distinct anodic peak at around 1.2 V in a nitrogen atmosphere; however, this peak is not present in an oxygen environment, corroborating the oxidation of HDP in O_2_ environments. Additionally, X-ray photoelectron spectroscopy (XPS) analysis revealed characteristic peaks of C-C, C-O, and C=O at binding energies of 284, 286, and 288 eV, respectively (Figure S1c). These spectral peaks confirm the existence of catechol groups under the N_2_ condition. Notably, the relative intensity of the C=O peak increased under oxygen-rich conditions, indicating enhanced oxidation of the polyphenolic HDP structure upon exposure to air [24,25]. Following the characterization of HDP nanoparticles, the HDP–PVA hydrogel was fabricated using PVA as the hydrogel matrix, with HDP was incorporated to impart O_2_-sensitive behavior and ROS scavenging functionality. To identify the optimal composition for sensor performance in nitrogen (N_2_) and oxygen (O_2_) environments, hydrogels were prepared using different weight ratios of PVA to HDP (100:0 [control], 100:1, and 100:5 wt%). As illustrated in Figure 1b, a visible color change between N_2_ and O_2_ conditions was evident only in the hydrogel with a 100:1 wt% ratio, indicating potential oxygen responsiveness. However, subsequent mechanical assessments revealed that this hydrogel was too brittle and mechanically weak for use as a functional sensor. In contrast, the hydrogel with a 100:5 wt% ratio demonstrated significantly enhanced mechanical performance, exhibiting tensile strengths of 0.08 ± 0.006 and 0.16 ± 0.001 MPa under N_2_ and O_2_, respectively (Figure 1c). This enhancement was notably higher than the 100:1 wt% hydrogel, which exhibited 0.05 ± 0.005 and 0.1 ± 0.011 MPa under the same conditions. The notably higher tensile strength under O_2_ conditions was attributed to the enhancement of the crosslinking density mediated by the oxidation of polyphenolic HDP into quinone moieties, thereby increasing the overall crosslinking density in the system [26,27,28]. Moreover, the 100:0 wt% hydrogel exhibited no significant changes, with 0.0277 ± 0.0062 and 0.0282 ± 0.0054 MPa under N_2_ and O_2_ conditions, respectively. However, this difference was not statistically significant. Furthermore, the scanning electron microscopy (SEM) imaging was performed to investigate the internal structure of the hydrogel. As illustrated in Figure 1d, under O_2_ conditions, the hydrogel samples with 100:1 and 100:5 wt% compositions exhibited a densely crosslinked internal microarchitecture with a smaller pore size arising from enhanced crosslinking owing to the oxygen–polyphenolic HDP interaction, thereby augmenting the overall crosslinking density of the hydrogel system [29,30,31]. In contrast, under N_2_ conditions, the same hydrogels displayed a loosely bound network with larger pores, due to the absence of HDP–oxygen interactions, thereby resulting in a softer and less crosslinked structure. Notably, the control hydrogel (100:0 wt%) did not exhibit any significant structural or mechanical differences between N_2_ and O_2_ environments. As shown in Figure S2, the pore area analysis further confirmed that the 100:1 and 100:5 wt% hydrogels showed a notable decrease in pore size under O_2_ conditions, consistent with the formation of a denser, more compact network structure mediated by HDP–oxygen crosslinking. Conversely, the control hydrogel (100:0 wt%) displayed no discernible changes in pore morphology, reinforcing the role of the polyphenolic HDP moiety in modulating the structural, morphological, and crosslinking characteristics of the fabricated hydrogel system.

### 2.2. Electrical and Wireless Output Analysis of the HDP–PVA Hydrogel

**Figure 2:**
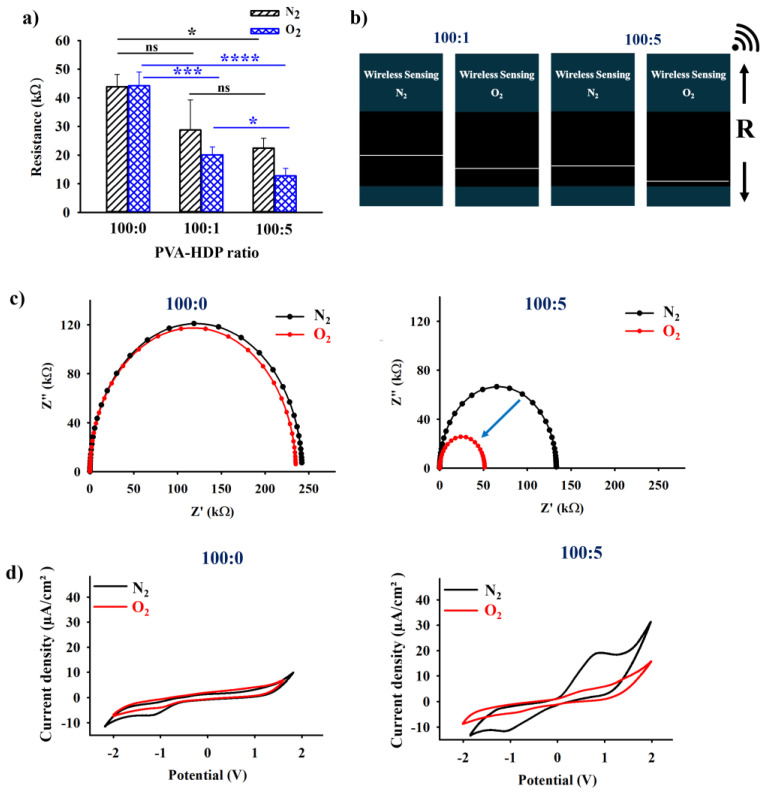
(**a**) Sourcemeter resistance, (**b**) wireless resistance analysis, (**c**) EIS Nyquist plot, and (**d**) cyclic voltammetry (CV) of HDP–PVA hydrogel in N_2_ and O_2_ conditions. Statistical analysis performed using variance (ANOVA) followed by Tukey’s multiple comparison test (* *p* < 0.05, *** *p* < 0.001, **** *p* < 0.0001, and ns indicates non-significant differences for *n* = 3 samples).

The electrochemical properties and influence of varying the HDP content in the fabricated HDP–PVA hydrogel were investigated using electrochemical impedance spectroscopy (EIS), source meter, and cyclic voltammetry (CV). As shown in Figure 2a, hydrogels with PVA/HDP ratios of 100:1 and 100:5 exhibited distinct differences in electrical resistance values in N_2_ and O_2_ environments. The significant decrease in resistance under O_2_ conditions can be attributed to the oxidation of the polyphenolic HDP moiety, which enhances the overall conductivity of the system through π–π stacking interactions [32,33,34,35]. To further validate these findings, wireless analysis was performed to investigate the distinct changes in the resistance of the hydrogel under different conditions, as indicated by the white line [36]. As illustrated in Figure S3, the wireless communication sensing system is composed of a voltage sensor, a 32-bit Arduino Uno R3.0 microcontroller, a Bluetooth module, the AppGosu communication system, and a smartphone. As shown in Figure 2b, the 100:5 wt% hydrogel sample displayed a more pronounced increase in electrical conductivity under O_2_ exposure than the 100:1 wt% sample. These results, combined with earlier mechanical evaluations, support the selection of the 100:5 wt% hydrogel for subsequent experiments.

The electrochemical impedance spectroscopy (EIS) was employed to assess the alterations in the electrical conductivity of the fabricated HDP–PVA hydrogel under N_2_ and O_2_ environments. The variations in semicircular diameter observed in the Nyquist plots reflect changes in charge transfer resistance, with a larger semicircle indicating higher resistance [37,38]. As shown in Figure 2c, the control hydrogel (100:0 wt%) exhibited negligible changes in impedance under N_2_ and O_2_ conditions. However, the hydrogel (100:5 wt%) displayed a significant reduction in impedance under O_2_ conditions, which corroborated a similar trend in the source meter and wireless resistance output as influenced by the polyphenolic HDP–oxygen interaction. In addition, cyclic voltammetry (CV) studies were conducted to investigate the redox activity of the polyphenolic HDP component at a scan rate of 50 mV/s from −2 to +2 V under N_2_ and O_2_ conditions. As shown in Figure 2d, the HDP–PVA hydrogel (100:5 wt%) displayed a distinct anodic peak of approximately 0.83 V (vs. Ag/AgCl) under N_2_ conditions, indicative of the oxidation potential of HDP [39,40,41]. In contrast, no such peak was observed under O_2_ conditions, implying that the HDP had already undergone oxidation in air. The control hydrogel (100:0 wt%) displayed no appreciable redox activity under either condition, confirming the pivotal role of HDP in the electrochemical responsiveness of the system.

### 2.3. Mechanical Compressive, Tensile Test, and ROS Scavenging Capability of the HDP–PVA Hydrogel

**Figure 3:**
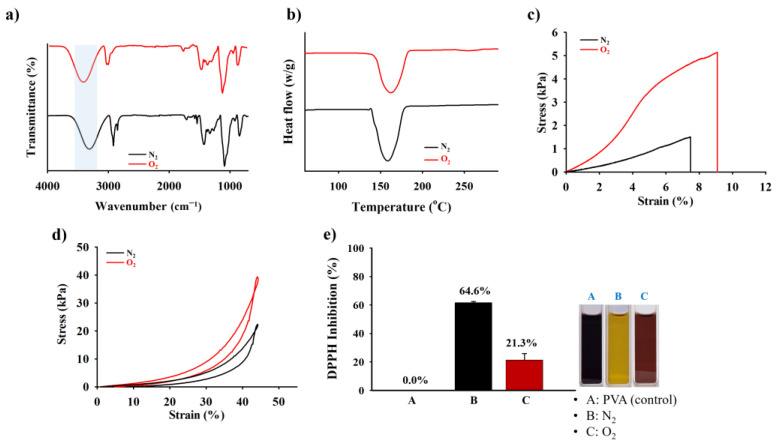
(**a**) FT-IR, (**b**) differential scanning calorimetry (DSC) thermogram, (**c**) stress vs. strain tensile test, (**d**) compression test, and (**e**) DPPH scavenging activity of HDP–PVA hydrogel (100:5 wt%) in N_2_ and O_2_ conditions.

Distinct changes in the internal mechanical structure of the HDP–PVA hydrogel under various environmental conditions were further investigated. As shown in Figure 3a, Fourier transform infrared (FT-IR) analysis revealed a notable shift in the -O-H peak from approximately 3272.6 cm^−1^ under N_2_ conditions to 3407.32 cm^−1^ in O_2_ conditions. This shift shows a change in hydrogen bonding and structural reorganization within the hydrogel matrix following oxygen exposure. The thermal degradation behavior of the HDP–PVA hydrogel was investigated using differential scanning calorimetry (DSC), which revealed a notable shift in the melting temperature (Tm) from 138.8 °C under an N_2_ atmosphere to 162.6 °C under an O_2_ atmosphere (Figure 3b). This increase in Tm under oxygenated conditions suggests enhanced thermal stability of the hydrogel system, which can be attributed to increased crosslinking density arising from oxidative interactions between HDP and oxygen. The changes in the internal structure were also reflected in the swelling behavior of the hydrogel. As illustrated in Figure S4, the hydrogel treated under O_2_ conditions exhibited reduced water uptake compared to that of the N_2_-exposed hydrogel. This phenomenon was likely because of the denser and more compact network structure resulting from the oxidative crosslinking of the polyphenolic HDP moieties, which enhanced the overall crosslinking density of the hydrogel [42,43]. Mechanical characterization further supported these findings. As shown in Figure 3c, the O_2_-treated hydrogel exhibited substantially enhanced mechanical performance, achieving a higher tensile strength of 5.15 kPa as compared to the N_2_-treated hydrogel with 1.53 kPa. Similarly, upon exposure to O_2_, the hydrogel displayed a relatively higher compression modulus owing to the comparatively tougher microstructural crosslinked network of the hydrogel matrix (Figure 3d). Moreover, the ultimate compressive stress (UCS)—defined as the maximum stress the material can withstand before fracture or failure—was measured at 39.52 kPa for O_2_-treated HDP–PVA hydrogels and 22.65 kPa for N_2_-treated hydrogels at 45% strain, indicating the superior structural integrity of the hydrogel in O_2_ environments. These findings indicate that the O_2_-treated hydrogel demonstrated a tougher and stiffer hydrogel, attributed to the denser crosslinking via oxidative polyphenolic chemistry. The reactive oxygen species (ROS)-scavenging capability of the HDP–PVA hydrogel was examined under N_2_ and O_2_ conditions using 2,2-diphenyl-1-picrylhydrazyl (DPPH) inhibition test. The hydrogel demonstrated superior antioxidant activity under N_2_ conditions, with a DPPH inhibition efficiency of 64.6%, compared to 21.3% under O_2_ conditions (Figure 3e). This enhanced activity in the N-treated sample is attributed to the presence of non-oxidized HDP groups, which remain available to neutralize free radicals [19,44,45]. Moreover, the images show a distinct color change in the DPPH solution upon treatment with the different samples (Figure 3e, inset) to further corroborate these results. The anti-interference test was conducted to assess the potential interferences from various stimuli, including NaCl, fetal bovine serum (FBS), FeCl_3_, and glucose, on the electrochemical performance of the hydrogel sensor (Figure S5). All the stimuli exhibited a notable increase in the electrical conductivity under O_2_ conditions, with the exception of ionic compounds such as NaCl and FeCl_3_, which inherently possess high ionic conductivity due to the presence of free ions and metal cations. Nevertheless, the HDP–PVA hydrogel treated with various stimuli could still display a notable difference in electrochemical output in N_2_ and O_2_ environments across all stimuli, thereby confirming the O_2_-responsive behavior of the fabricated hydrogel sensor.

### 2.4. In Vitro Rheological, Mechanical Compressive Test, and Electrical Analysis of the HDP–PVA Hydrogel

The in vitro cancer-sensing capability of the fabricated HDP–PVA hydrogel was examined by investigating alterations in its mechanophysical properties and electroconductivity characteristics. CHO-K1 cells were used as normal cells, and HeLa and B16F10 cells were used as cancer cell models. As widely reported in the literature, cancer cells often exhibit a hypoxic microenvironment owing to uncontrolled proliferation [46,47]. To confirm this, intracellular hypoxia was visualized using BioTracker 520 hypoxia green dye [48]. As shown in Figure S6, strong green fluorescence was observed in HeLa and B16F10 cells, confirming their hypoxic state, whereas CHO-K1 cells showed minimal fluorescence, which is consistent with normoxic conditions.

**Figure 4:**
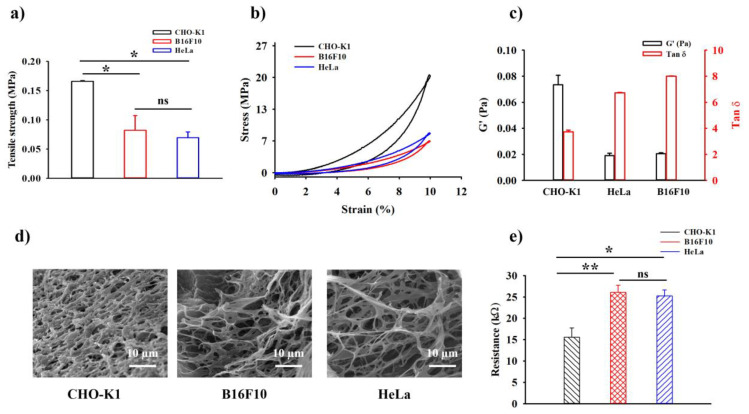
(**a**) Tensile test, (**b**) compression analysis, (**c**) rheological G’ (Pa) vs. Tan δ, (**d**) SEM images, and (**e**) sourcemeter resistance of cell-treated HDP–PVA hydrogel. Cell concentration, 1 × 10^5^ cells/mL. Statistical analysis performed using variance (ANOVA) followed by Tukey’s multiple comparison test (* *p* < 0.05, ** *p* < 0.01, and ns indicates non-significant differences for *n* = 3 samples).

To investigate the selectivity of the fabricated HDP–PVA hydrogel in the cancer microenvironment, the hydrogels were treated with either normal or cancer cells. As shown in Figure 4a, the CHO-K1 cell-treated hydrogel displayed significantly higher tensile strength of 0.17 ± 0.001 MPa as compared to those treated with HeLa (0.08 ± 0.025 MPa) and B16F10 (0.07 ± 0.009 MPa) cells. This enhancement in mechanical strength was attributed to the increased crosslinking density driven by HDP oxidation under normoxic conditions, which reinforced the overall structural integrity of the hydrogel. The stress vs. strain compressive modulus test followed a similar trend, where CHO-K1-treated hydrogel displayed a higher compressive stress (Figure 4b). Rheological analysis further supported these findings. The hydrogel treated with CHO-K1 cells exhibited an increased storage modulus (G’) and reduced loss tangent (tan δ), indicating a more elastic and stable network structure resulting from oxygen-mediated HDP crosslinking (Figure 4c). In contrast, HeLa and B16F10 cell-treated hydrogels displayed lower storage modulus (G’) values and higher tan δ, indicating a softer nature of the hydrogel system. The SEM images further elucidated the change in the internal crosslinking structure of the hydrogel matrix in normal and cancer cell microenvironments. As shown in Figure 4d, the CHO-K1 cell-treated hydrogel displayed a rather compact and densely crosslinked microstructure, whereas the hydrogels exposed to cancer cells appeared more porous and loosely connected, highlighting limited crosslinking under hypoxic conditions. These observations confirmed the role of HDP oxidation in enhancing the overall internal structural architecture of the hydrogel in normoxic environments. The change in the electroconductivity was further investigated using a source meter. As evident from Figure 4e, the normal cell-treated hydrogel displayed higher conductivity, likely because of π–π stacking interactions facilitated by the oxidized HDP moieties. In contrast, the cancer cell-treated hydrogels showed superior resistance, indicating that HDP oxidation had no influence on the system. To evaluate the analytical performance of the HDP–PVA hydrogel sensor, a linear calibration curve was generated by plotting the relative resistance (Δ*R*) against cell concentration of CHO-K1 and HeLa, ranging from 10^1^ to 10^5^ cells/mL (Figure S7). The limit of detection (LOD) was obtained as 52.46 and 43.07 cells/mL for CHO-K1 (*R*^2^ = 0.9998) and HeLa (*R*^2^ = 0.9994) cells, implying the sensitivity of the fabricated biosensor towards cancer cells.

### 2.5. In Vitro Biocompatibility and ROS-Scavenging Assessment of the HDP–PVA Hydrogel

**Figure 5:**
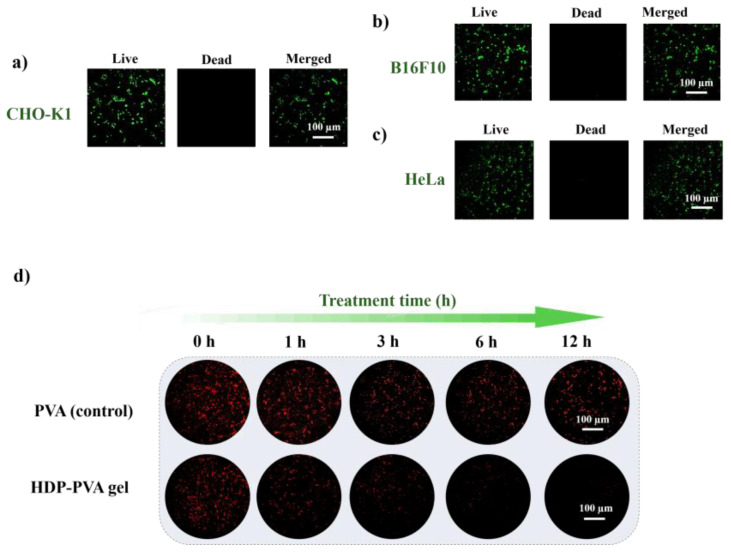
(**a**–**c**) Live and dead assay and (**d**) time-dependent ROS-scavenging assessment of the B16F10 cell-treated HDP–PVA hydrogel. Cell concentration 1 × 10^5^ cells/mL.

The biocompatibility of the HDP–PVA hydrogel was evaluated using a live and dead staining assay. As illustrated in Figure 5a–c, the cells displayed good viability upon treatment with the HDP–PVA hydrogel, as indicated by the bright green fluorescence, indicating high viability and good cytocompatibility. This favorable cell response suggests that the HDP–PVA hydrogel is well-tolerated in a biological environment. The cytotoxicity of the hydrogel was further assessed using an MTT assay with CHO-K1 and HeLa cells. The MTT assay is a simple method that is widely used to examine the toxicity of biomaterials [49,50]. As shown in Figure S8, the HDP–PVA hydrogel exhibited higher proliferation, indicating favorable biocompatibility. Given the known antioxidant properties of polyphenolic compounds, the ROS-scavenging ability of the HDP–PVA hydrogel was further evaluated using dihydroethidium (DHE) staining, which is a fluorescent probe for reactive oxygen species. As shown in Figure 5d, the HDP–PVA hydrogel treated with B16F10 cells demonstrated significant ROS-scavenging capability with increasing incubation time, as evidenced by a progressive reduction in the red fluorescence intensity of the DHE dye. In contrast, the PVA-only hydrogel exhibited no noticeable scavenging effect, suggesting the specific contribution of the HDP component. Similarly, as shown in Figure S9a, the CHO-K1 cell-treated hydrogel displayed no scavenging activity, in contrast to the HeLa cell-treated hydrogel, which displayed a notable decrease in DHE fluorescence (Figure S9b). Additionally, the expression levels of inflammation-associated oncogenic markers were analyzed using real-time polymerase chain reaction (RT-PCR), focusing on interleukin-1β (IL-1β) and nuclear factor kappa-light-chain-enhancer of activated B cells (NF-κB), both of which play pivotal roles in facilitating cancer cell proliferation and survival [51,52]. As shown in Figure S10, a significant downregulation in the expression of IL-1β and NF-κB was observed after treatment with the HDP–PVA hydrogel, thereby highlighting its potential for therapeutic application in cancer treatment. These results highlight the functional antioxidant capability of the fabricated HDP–PVA hydrogel and suggest its potential for therapeutic and diagnostic applications in cancer detection platforms.

## 3. Conclusions

In this study, we developed an O_2_-specific HDP–PVA hydrogel biosensor capable of distinguishing between cancerous and normal cellular microenvironments based on distinct mechanophysical and electrochemical responses. The presence of an oxygen-sensitive polyphenolic HDP moiety in the hydrogel modulated the mechanophysical and electrochemical characteristics of the biosensor, exhibiting oxygen-induced crosslinking and enhanced tensile strength, structural compactness, and conductivity under normoxic conditions. Electrochemical analyses were confirmed by increased conductivity under O_2_ conditions using HDP oxidation and π–π stacking interactions. In vitro studies have shown selective mechanoelectrical responses when exposed to normal (CHO-K1) versus cancer cells (HeLa, B16F10), as evidenced by the differential physical, rheological, and microstructural properties. Moreover, the HDP–PVA hydrogel demonstrated excellent ROS-scavenging activity, highlighting its potential as a functional platform for applications in cancer diagnostics and the monitoring of hypoxia-related pathologies.

## 4. Materials and Methods

### 4.1. Materials and Characterizations

Polyvinyl alcohol (Mw = 125,000 g/mol), DPPH, and phosphate-buffered saline (PBS) were purchased from Sigma-Aldrich (Seoul, Republic of Korea). [1,1′-biphenyl]-2,2′,4,4′,5,5′-hexaol (HDP) was purchased from FNB Tech (Pittsburgh, PA, USA). Dulbecco’s modified Eagle’s medium (DMEM), minimum essential medium (MEM), Roswell Park Memorial Institute (RPMI-1640), penicillin–streptomycin, trypsin–ethylenediaminetetraacetic acid (0.05% *w*/*v* trypsin-EDTA 1×), horse serum (10%), and fetal bovine serum (FBS, 5%) were acquired from Gibco BRL (Waltham, MA, USA). The CHO-K1, HeLa, and B16-F10 cell lines were procured from the Korean Cell Line Bank (Seoul, Republic of Korea). Propidium iodide (PI) and calcein AM were purchased from Invitrogen (Waltham, MA, USA). DHE and BioTracker^TM^ 520 green hypoxia dyes were purchased from Sigma-Aldrich (St. Louis, MO, USA). 3-(4,5-dimethylthiazol-2-yl)-2,5-diphenyltetrazolium bromide (MTT) was purchased from Sigma Aldrich, Republic of Korea.

Fourier transform infrared spectroscopy (FTIR) was performed using an FTIR spectrometer (Thermo Scientific, Madison, WI, USA) in the scan range 600–4000 cm^−1^ to identify and confirm the presence of specific functional groups and chemical bonds within the samples. UV-visible (UV-Vis) spectra were obtained using an Optizen 2020UV spectrometer (Mecasys, Daejeon, Republic of Korea) to examine the absorbance characteristics and electronic transitions. Differential scanning calorimetry (DSC, TA Instruments, New Castle, DE, USA) was performed using a DSC2910 differential thermal analyzer with a heating rate of 10 °C/min ranging 25–300 °C under a nitrogen atmosphere. DSC was used to analyze the thermal properties of the materials under controlled heating conditions. Scanning electron microscopy (SEM) images were obtained using a JSM-6700F microscope (JEOL, Tokyo, Japan) to examine the surface morphology and microstructural features such as porosity, texture of the sample. The X-ray photoelectron spectroscopy (XPS) results were obtained using an Omicrometer ESCALAB (XPS, PHI Quantera-Ⅱ, Ulvac-PHI, Chigasaki, Japan). Confocal laser scanning microscopy (CLSM) images were recorded using a Nikon ECLIPSE Ti2-E (Nikon corporation, Tokyo, Japan) confocal microscope. CLSM was used to visualize the stained cells to assess the live and dead assay and ROS staining. The electrochemical properties of the materials were assessed using an electrochemical impedance spectrometer (EIS; CS350, CorrTest Instrument, Wuhan, China), a sourcemeter (Keithley 2450, Tektronix, Beaverton, OR, USA), and cyclic voltammetry (CV) (CorrTest Electrochemical Workstation, Wuhan, China) to evaluate the electrical conductive behavior under various conditions. The rheology was assessed using a HAAKE MARS modular advanced rheometer with a 20 mm parallel plate geometry to determine the viscoelastic properties of the samples, including deformation behavior under applied stress. Mechanical testing, such as stress–strain fracture and compression tests, was conducted using a Universal Testing Machine (UTM, SurTA 1A, Chemilab Co., Gimcheon, Republic of Korea) to assess the mechanical strength and elasticity of the samples. An Arduino Uno microcontroller (ATmega328P Processor) (Zeno, Busan, Republic of Korea) served as a sensor for the wireless monitoring of real-time data presented on a smartphone. The AppGosu software (Ver. 2) communicated wirelessly between the smartphone and the Arduino Uno.

### 4.2. Fabrication of O_2_-Responsive HDP–PVA Hydrogel

To prepare the HDP–PVA hydrogel, different weight ratios of PVA/HDP (100:0, 100:1, 100:5 wt%) were prepared by dispersing HDP into the PVA solution and stirring until homogenous at room temperature, while maintaining the N_2_ condition to prevent HDP oxidation. The resultant solution was poured into a hydrogel mold and subjected to freezing–thawing for 3× cycles under inert conditions. The resultant HDP–PVA hydrogel was air-sealed and stored for further use. The hydrogel was treated under N_2_ (~1% O_2_) and O_2_ conditions.

### 4.3. Physical and Mechanical Assessment of the HDP–PVA Hydrogel

The hydrogel was treated under N_2_ conditions (~1% O_2_) to mimic the hypoxic cancer microenvironment, whereas normoxic conditions were maintained in an ambient atmosphere (approximately 21% O_2_). Hydrogel samples with dimensions of 20 × 10 × 1.5 mm^3^ (length × breadth × thickness) were evaluated using a SurTA4 universal testing machine (UTM) (UTM, SurTA 1A, Chemilab Co., Gimcheon, Republic of Korea). Each sample was fastened at both ends and stretched at a steady rate of 1 mm/s. For the compression test, the hydrogel sample with dimensions (1 × 1 × 1) cm^3^ was subjected to a compression at a constant speed of 5 mm/min, resulting in a compression rate of 8.33 × 10^3^ mm/s.

### 4.4. Evaluation of Electrochemical and Wireless Analysis of HDP–PVA Hydrogel

Distinct changes in the electrical properties of the fabricated hydrogels were assessed using electrochemical impedance spectroscopy (EIS), cyclic voltammetry (CV), and a source meter. The EIS system was set up at 0 V against an open-circuit potential (OCP) frequency ranging 0.1–10 kHz using a single electrode serving as both the reference and counter electrodes. The direct current (DC) resistance was measured using a Keithley 2450 source meter, a 1 V BIAS voltage, and a 0.01 s measure delay. Compliance was maintained at 1 V by using a two-electrode DC system (*n* = 3 for each sample). Cyclic voltammetry (CV) was performed using the HDP–PVA hydrogel as the working electrode, Ag/AgCl as the reference electrode, and a platinum wire as the counter electrode. The measurements were taken at a scan rate of 50 mV/s across a potential range of −2 to +2 V relative to the Ag/AgCl reference. For wireless monitoring, the hydrogel was attached to a circuit that included an Arduino Uno R3.0 microprocessor and a Bluetooth module. The signal was wirelessly transferred to a smartphone, where the AppGosu program allowed the real-time display of the output using a smartphone [36].

### 4.5. Evaluation of Radical Scavenging Performance of the HDP–PVA Hydrogel

The radical scavenging capability of the fabricated HDP–PVA hydrogel was investigated using HDP–PVA hydrogel immersed in 0.1 mM DPPH solution and incubated at 37 °C for 45 min under N_2_ and O_2_ conditions. The absorbance of the resulting solution was measured using ultraviolet-visible (UV-Vis) spectroscopy [53]. The DPPH inhibition was calculated using the following equation:

DPPH inhibition (%) = (Astd−AsamAstd)×100%

where A_std_ = absorbance of DPPH standard and A_sam_ = absorbance of the sample.

### 4.6. In Vitro Analysis of the Mechanical and Electrical Properties of HDP–PVA Hydrogel

The in vitro evaluation was performed using CHO-K1 (normal cells), HeLa, and B16F10 (cancer cells) as models. First, the cells were cultured in 25 mm^2^ T-flasks at 37 °C in a humid atmosphere with 5% CO_2_ until they reached 70–80% confluence, either under hypoxic (approximately 1% O_2_) or normoxic (approximately 21% O_2_) conditions. A chamber containing 1% O_2_, 5% CO_2_, and 94% N_2_ was used to simulate hypoxic conditions, whereas a conventional incubator was used to simulate the normoxic environment. A 200 µL cell suspension (1 × 10^5^ cells/mL) was seeded into the fabricated HDP–PVA hydrogel and incubated for 12 h under both conditions. After incubation, the hydrogel was rinsed with PBS (pH 7.4) to remove loosely attached cells, and its electrochemical and mechanical characteristics were promptly assessed. The mechanical properties, such as tensile and compressive tests, and electrical properties were analyzed following the previously mentioned methods.

### 4.7. In Vitro ROS Scavenging Capability of HDP–PVA Hydrogel

The in vitro ROS-scavenging capability of the fabricated HDP–PVA hydrogel was assessed by seeding cells onto the hydrogel sample at a density of 1 × 10^5^ cells/mL at 37 °C in a 5% CO_2_ humid atmosphere under normoxic (~21% O_2_) and hypoxic (approximately 1% O_2_) conditions. After different intervals of treatment, the cells were washed with PBS (pH 7.4), followed by an incubation of 500 µM (50 µL) of DHE (red fluorescence) dye for 30 min at 37 °C. The cells were imaged using a confocal laser scanning microscope (CLSM) (Nikon ECLIPSE Ti2-E (Nikon corporation, Tokyo, Japan).

### 4.8. Live and Dead Assessment of HDP–PVA Hydrogel

For the live and dead assay, a 200 µL cell suspension (1 × 10^5^ cells/mL) was added to the HDP–PVA hydrogel and incubated for 12 h at 37 °C in a humidified environment with 5% CO_2_. After incubation, PBS (pH 7.4) was used to remove loosely bound cells. The cells were centrifuged at 1500 rpm for 3 min. After removing the supernatant, 100 µL of calcein AM (green stain for live cells) was added and incubated for 15 min, followed by the addition of 50 µL of PI (red stain for dead cells). CLSM was used at 20× magnification to observe the stained cells.

### 4.9. MTT Assay

For the MTT assay, HeLa (cancer cell) and CHO-K1 (normal cell) were seeded into 96-well plates at a density of 5 × 10^5^ cells/mL (200 μL per well) and cultured for 24 h at 37 °C in a 5% CO_2_ atmosphere. Following medium removal, cells were exposed to treatment with HDP–PVA hydrogel for 12 h. Subsequently, the medium was replaced with 200 μL of thiazolyl blue solution (0.5 mg/mL), and cells were incubated for an additional 4 h. After discarding the thiazolyl blue solution, 200 μL of MTT solubilization reagent was added to each well. The plates were agitated for 15 min, and absorbance was measured at 570 nm using a microplate reader (BMG LABTECH, Ortenberg, Germany). Wells containing only cells without treatment served as controls.

### 4.10. RNA Isolation and Quantitative Real-Time (qRT)-PCR

RNA was isolated using RNAiso plus (#9109, Takara, Kyoto, Japan) and reverse transcribed using 5X All-In-One RT Master Mix (RR036A PrimeScript™ RT Master Mix (Perfect Real Time)) (Takara, Kyoto, Japan). Real time qRT-PCR was performed using QuantStudio 3 Real- Time PCR (Thermo Fisher scientific, Waltham, MA, USA). All the qRT-PCR reactions were performed using RR830A TB Green^®^ Premix Ex Taq™ II FAST qPCR, in a StepOne plus real time PCR machine (Applied Biosystem, Waltham, MA, USA). The primers were as follows: β-actin: Forward: 5′-GGC ATC CTC ACC CTG AAG TA-3′, Reverse: 3′-AGG TGT GGt GCC AGA TTT TC-5′, NF-kB: Forward: 5′-CCT GGA TGA CTC TTG GGA AA-3′, Reverse: 3′-TCA GCC AGC TGT TTC ATG TC-5′, IL-1b: Forward: 5′-TCC AGG ATG AGG ACA TGA GCA C-3′, Reverse: 3′-GAA CGT CAC ACA CCA GCA GGT TA-5′.

## Data Availability

Dataset available upon request from the authors.

## Supplementary Materials

The following supporting information can be downloaded at: https://www.mdpi.com/article/10.3390/gels11080566/s1, Figure S1: (a) UV-Vis spectra, CV, and XPS spectra of HDP nanoparticles. Figure S2: Pore size analysis of HDP–PVA hydrogel. Figure S3: Wireless sensing communication setup. Figure S4: Swelling ratio profile of HDP–PVA hydrogel. Figure S5: Anti-interference test of HDP–PVA hydrogel. Figure S6: Images of BioTrackerTM 520 hypoxia dye signals on cells. Figure S7: Limit of detection (LOD) data. Figure S8: MTT assay. Figure S9: Time-dependent ROS-scavenging activity of PVA and HDP–PVA hydrogel treated with CHO-K1 cells, and HeLa cell lines. Figure S10: Gene expression data.

## References

[1] Chen P.S., Chiu W.T., Hsu P.L., Lin S.C., Peng I.C., Wang C.Y., Tsai S.J. (2020). Pathophysiological Implications of Hypoxia in Human Diseases. J. Biomed. Sci..

[2] Khan B.D., Nhan T.N.T., Chau H.N.M., Nhi N.T.Y. (2022). Roles of Hypoxia in Tumor Progression and Novel Strategies for Cancer Treatment. Biomed. Res. Ther..

[3] Lewis D.M., Blatchley M.R., Park K.M., Gerecht S. (2017). O_2_-Controllable Hydrogels for Studying Cellular Responses to Hypoxic Gradients in Three Dimensions in Vitro and in Vivo. Nat. Protoc..

[4] Yang G., Shi R., Zhang Q. (2020). Hypoxia and Oxygen-Sensing Signaling in Gene Regulation and Cancer Progression. Int. J. Mol. Sci..

[5] Lee K.K., Go K., Lee E., Kim H., Kim S., Kim J., Chae M.S., Jeong J. (2025). Multifunctional Hydrogels for Advanced Cancer Treatment: Diagnostic Imaging and Therapeutic Modalities. Gels.

[6] Ding K., Liao M., Wang Y., Lu J.R. (2025). Advances in Composite Stimuli-Responsive Hydrogels for Wound Healing: Mechanisms and Applications. Gels.

[7] Ahmad Z., Salman S., Khan S.A., Amin A., Rahman Z.U., Al-Ghamdi Y.O., Akhtar K., Bakhsh E.M., Khan S.B. (2022). Versatility of Hydrogels: From Synthetic Strategies, Classification, and Properties to Biomedical Applications. Gels.

[8] Chen J., Pan C., Gao Y., Chen Q., An X., Liu Z. (2024). Reactive Oxygen Species Scavenging Injectable Hydrogel Potentiates the Therapeutic Potential of Mesenchymal Stem Cells in Skin Flap Regeneration. ACS Appl. Mater. Interfaces.

[9] Liu X., Chen X., Fei Y., Zhang J., Yue O., Wang X., Jiang H. (2025). Locally Injectable, ROS-Scavenging, and ROS-/PH-Responsive Polymeric-Micelles-Embedded Hydrogels for Precise Minimally Invasive and Long-Lasting Rheumatoid Therapy. Adv. Healthc. Mater..

[10] Zhang J., Zhong J., Liang B., Liu W., Zhang H., Wang T., Huang L., Yang L., Gu Z., Li Y. (2025). Robust Injectable Hydrogels for Hemophilic Arthropathy via Anti-Inflammation, Iron Removal and Cartilage Protection. Adv. Funct. Mater..

[11] Xu Y., Zheng S., Tang Z., Zhong Q., Chen R., Wang P., Fu J., Xie J., Ning Y., Lei M. (2024). Injectable, Oxygen-Releasing, Thermosensitive Hydrogel Promotes Vascularized Bone Formation with Prolonged Oxygen Delivery and Improved Osteoinductivity. Mater. Today Bio.

[12] Dey A., Roy K., Subba S.H., Lee G., Park S.Y. (2025). MXene/Polymer Dot-Decorated Flexible Sensor for Cancer Cell-Responsive Hydrogel with Tunable Elastic Modulus, Porosity, and Conductivity. Talanta.

[13] Moon J.R., Kim J.H. (2008). Biodegradable Thermo- and PH-Responsive Hydrogels Based on Amphiphilic Poly-Aspartamide Derivatives Containing N,N-Diisopropylamine Pendants. Macromol. Res..

[14] Völlmecke K., Afroz R., Bierbach S., Brenker L.J., Frücht S., Glass A., Giebelhaus R., Hoppe A., Kanemaru K., Lazarek M. (2022). Hydrogel-Based Biosensors. Gels.

[15] Liu D., Huyan C., Wang Z., Guo Z., Zhang X., Torun H., Mulvihill D., Xu B., Chen F. (2023). Conductive Polymer Based Hydrogels and Their Application in Wearable Sensors: A Review. Mater. Horiz..

[16] Kougkolos G., Golzio M., Laudebat L., Valdez-Nava Z., Flahaut E. (2023). Hydrogels with Electrically Conductive Nanomaterials for Biomedical Applications. J. Mater. Chem. B.

[17] Rao K.M., Uthappa U.T., Kim H.J., Han S.S. (2023). Tissue Adhesive, Biocompatible, Antioxidant, and Antibacterial Hydrogels Based on Tannic Acid and Fungal-Derived Carboxymethyl Chitosan for Wound-Dressing Applications. Gels.

[18] Dong L., Jia R., Liu Z., Aiyiti W., Shuai C., Li Z., Fu Q., Li X. (2024). Tannic Acid Based Multifunctional Hydrogels with Mechanical Stability for Wound Healing. Colloids Surf. B Biointerfaces.

[19] Kim T.M., Won H.J., Yang J.H., Jo H., Kim A.H., Nam D., Kim S.G., Jin E.J., Bae H.J., Park S.Y. (2023). Multicolor Hair Dyeing with Biocompatible Dark Polyphenol Complex-Integrated Shampoo with Reactive Oxygen Species Scavenging Activity. Biomimetics.

[20] Zhou Y., Zheng J., Li Y., Xu D.P., Li S., Chen Y.M., Li H. (2016). Bin Natural Polyphenols for Prevention and Treatment of Cancer. Nutrients.

[21] Rahman Khan M.M., Rumon M.M.H. (2025). Synthesis of PVA-Based Hydrogels for Biomedical Applications: Recent Trends and Advances. Gels.

[22] Liang X., Zhong H.-J., Ding H., Yu B., Ma X., Liu X., Chong C.-M. (2024). Polyvinyl alcohol (PVA)-based hydrogels: Recent progress in fabrication, properties, and multifunctional applications. Polymers.

[23] Hou J., Li C., Guan Y., Zhang Y., Zhu X.X. (2015). Enzymatically Crosslinked Alginate Hydrogels with Improved Adhesion Properties. Polym. Chem..

[24] Xia Q., Liang Y., Cao A., Cao Y., Cai L. (2024). Preparation and Characterization of PH-Responsive Metal-Polyphenol Structure Coated Nanoparticles. Food Sci. Hum. Wellness.

[25] Tian Z., Wu G., Libby M., Wu K., Jeong K.J., Kim Y.J. (2023). Synthesis of Biologically Derived Poly(Pyrogallol) Nanofibers for Antibacterial Applications. J. Mater. Chem. B.

[26] Han N., Xu Z., Cui C., Li Y., Zhang D., Xiao M., Fan C., Wu T., Yang J., Liu W. (2020). A Fe^3+^-Crosslinked Pyrogallol-Tethered Gelatin Adhesive Hydrogel with Antibacterial Activity for Wound Healing. Biomater. Sci..

[27] Subba S.H., Jiang S., Jin E.J., Park S.Y. (2025). Hypoxia-Sensitive Smart Hydrogel Biosensor for Distinct Mechanical and Electrical Signals with Muscle Ischemia Regeneration. Adv. Funct. Mater..

[28] Shen J., Gao G., Liu X., Fu J. (2015). Natural Polyphenols Enhance Stability of Crosslinked UHMWPE for Joint Implants. Clin. Orthop. Relat. Res..

[29] Lee F., Chung J.E., Xu K., Kurisawa M. (2015). Injectable Degradation-Resistant Hyaluronic Acid Hydrogels Cross-Linked via the Oxidative Coupling of Green Tea Catechin. ACS Macro Lett..

[30] Gijutsu S., Kenkyujo S., Chuo T., Technologies S. (2019). Catechol-Functionalized Hydrogels: Biomimetic Design, Adhesion Mechanism, and Biomedical Applications. Chem. Soc. Rev..

[31] An S., Jeon E.J., Kim M., Han S.Y., Song Y.S., Jeon J., Park J.U., Cho S.W. (2024). Endomysium-Permeable Muscle Extracellular Matrix Composite Hydrogel for Promoting Functional Muscle Recovery in Muscle Atrophy. Chem. Eng. J..

[32] Shin M., Park E., Lee H. (2019). Plant-Inspired Pyrogallol-Containing Functional Materials. Adv. Funct. Mater..

[33] Son E.J., Kim J.H., Kim K., Park C.B. (2016). Quinone and Its Derivatives for Energy Harvesting and Storage Materials. J. Mater. Chem. A.

[34] Han X., Kong H., Chen T., Gao J., Zhao Y., Sang Y., Hu G. (2021). Effect of π–π Stacking Interfacial Interaction on the Properties of Graphene/Poly(Styrene-b-Isoprene-b-Styrene) Composites. Nanomaterials.

[35] Su Z., Wang H., Tian K., Huang W., Xiao C., Guo Y., He J., Tian X. (2018). The Combination of π-π Interaction and Covalent Bonding Can Synergistically Strengthen the Flexible Electrical Insulating Nanocomposites with Well Adhesive Properties and Thermal Conductivity. Compos. Sci. Technol..

[36] Jo H.J., Shit A., Jhon H.S., Park S.Y. (2020). Highly Sensitive Non-Enzymatic Wireless Glucose Sensor Based on Ni–Co Oxide Nanoneedle-Anchored Polymer Dots. J. Ind. Eng. Chem..

[37] Lemaitre L., Moors M., Van Peteghem A.P. (1983). The Estimation of the Charge Transfer Resistance by Graphical Analysis of Inclined Semicircular Complex Impedance Diagrams. J. Appl. Electrochem..

[38] Laschuk N.O., Easton E.B., Zenkina O.V. (2021). Reducing the Resistance for the Use of Electrochemical Impedance Spectroscopy Analysis in Materials Chemistry. RSC Adv..

[39] Behboodi-Sadabad F., Zhang H., Trouillet V., Welle A., Plumeré N., Levkin P.A. (2017). UV-Triggered Polymerization, Deposition, and Patterning of Plant Phenolic Compounds. Adv. Funct. Mater..

[40] Ball V. (2022). Electrodeposition of Pyrogallol versus Pyrocatechol Using Cyclic Voltammetry and Chronoamperometry. J. Electroanal. Chem..

[41] Astaf’eva T.V., Arsenyev M.V., Rumyantcev R.V., Fukin G.K., Cherkasov V.K., Poddelsky A.I. (2020). Imine-Based Catechols and o-Benzoquinones: Synthesis, Structure, and Features of Redox Behavior. ACS Omega.

[42] Randolph C., Lahive C.W., Sami S., Havenith R.W.A., Heeres H.J., Deuss P.J. (2018). Biobased Chemicals: 1,2,4-Benzenetriol, Selective Deuteration and Dimerization to Bifunctional Aromatic Compounds. Org. Process. Res. Dev..

[43] Chen C., Li D., Yano H., Abe K. (2019). Bioinspired Hydrogels: Quinone Crosslinking Reaction for Chitin Nanofibers with Enhanced Mechanical Strength via Surface Deacetylation. Carbohydr. Polym..

[44] Jia Z., Gong J., Zeng Y., Ran J., Liu J., Wang K., Xie C., Lu X., Wang J. (2021). Bioinspired Conductive Silk Microfiber Integrated Bioelectronic for Diagnosis and Wound Healing in Diabetes. Adv. Funct. Mater..

[45] Kapiszewska M., Soltys E., Visioli F., Cierniak A., Zajac G. (2005). The protective ability of the Mediterranean plant extracts against the oxidative DNA damage. The role of the radical oxygen species and the polyphenol content. J. Physiol. Pharmacol. Suppl..

[46] Luo N. (2022). Editorial: Tumor Microenvironment in Cancer Hallmarks and Therapeutics. Front. Mol. Biosci..

[47] Petrova V., Annicchiarico-Petruzzelli M., Melino G., Amelio I. (2018). The Hypoxic Tumour Microenvironment. Oncogenesis.

[48] D’Aiuto N., Hochmann J., Millán M., Di Paolo A., Bologna-Molina R., Sotelo Silveira J., Arocena M. (2022). Hypoxia, Acidification and Oxidative Stress in Cells Cultured at Large Distances from an Oxygen Source. Sci. Rep..

[49] Ghasemi M., Turnbull T., Sebastian S., Kempson I. (2021). The Mtt Assay: Utility, Limitations, Pitfalls, and Interpretation in Bulk and Single-Cell Analysis. Int. J. Mol. Sci..

[50] Caló E., Barros J., Ballamy L., Khutoryanskiy V.V. (2016). Poly(Vinyl Alcohol)-Gantrez^®^ AN Cryogels for Wound Care Applications. RSC Adv..

[51] Scholz C.C., Cavadas M.A.S., Tambuwala M.M., Hams E., Rodríguez J., Von Kriegsheim A., Cotter P., Bruning U., Fallon P.G., Cheong A. (2013). Regulation of IL-1β-Induced NF-kB by Hydroxylases Links Key Hypoxic and Inflammatory Signaling Pathways. Proc. Natl. Acad. Sci. USA.

[52] Xia Y., Shen S., Verma I.M. (2014). NF-ΚB, an Active Player in Human Cancers. Cancer Immunol. Res..

[53] Min Kim T., Ryplida B., Lee G., Young Park S. (2023). Cancer Cells Targeting H_2_O_2_-Responsive MXene-Integrated Hyaluronic Acid Polymer Dots Coated Sensor. J. Ind. Eng. Chem..

