# Supplementary material for: O2-Generated Electrical and Mechanical Properties of Polyphenol-Mediated Hydrogel Sensor
Source: Gels. 2025 Jul 22;11(8):566. doi: 10.3390/gels11080566 (PMC12385903; doi:10.3390/gels11080566)
Supplement: Supplementary file 1 [file gels-11-00566-s001.zip › gels-3736083-supplementary.pdf]

Supporting Information

# $O_2$-generated electrical and mechanical properties of polyphenol-mediated hydrogel sensor

*Sunu Hangma Subba[1#], A Hyeon Kim[2#], Anneshwa Dey[1], Byung Chan Lee[2*], Sung Young Park[1,3*]*

[1]Department of IT and Energy Convergence, Korea National University of Transportation, Chungju 27469, Republic of Korea

[2]Department of Chemical & Biological Engineering, Korea National University of Transportation, Chungju 27469, Republic of Korea

[3]Department of Environmental Engineering, Korea National University of Transportation, Chungju 27469, Republic of Korea

*Corresponding authors:

E-mail: bclee@ut.ac.kr (B. C. Lee), parkchem@ut.ac.kr (S.Y. Park)

[#]These authors equally contributed to this work.

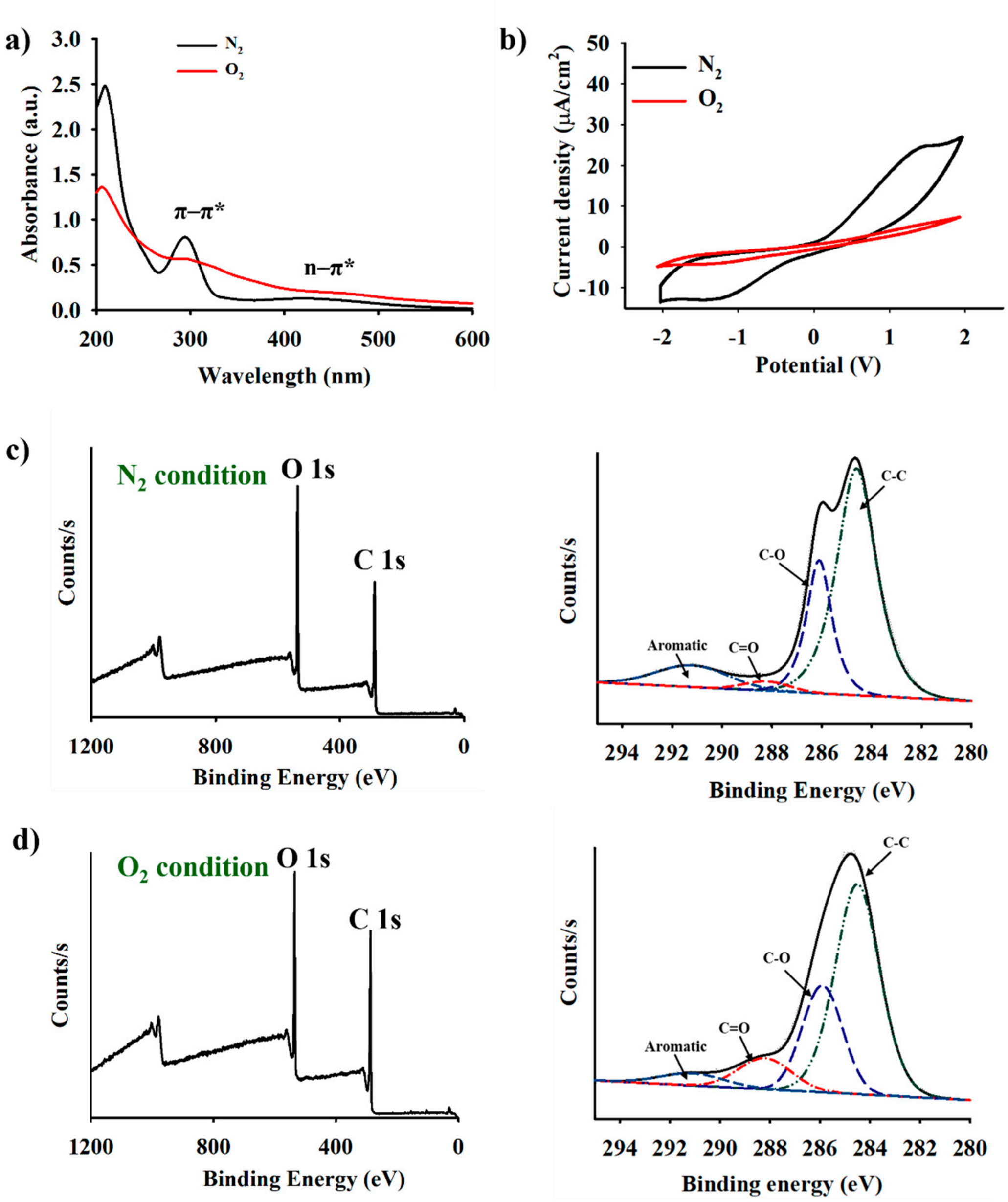


**Figure S1: a)** UV-vis spectra, **b)** cyclic voltammetry (CV), and XPS spectra of HDP nanoparticles under **c)** $N_2$ and **d)** $O_2$ conditions.

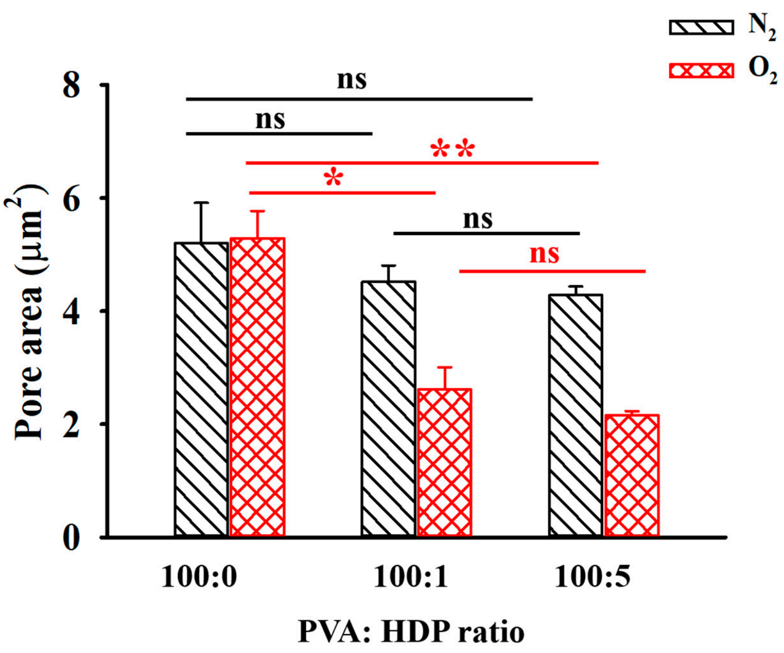


**Figure S2:** Pore area analysis of PVA: HDP hydrogel measured using ImageJ under $N_2$ and $O_2$ conditions. Statistical analysis performed using variance (ANOVA) followed by Tukey's multiple comparison test (*$p < 0.05$, **$p < 0.01$, and ns indicates non-significant differences for $n = 3$ samples).

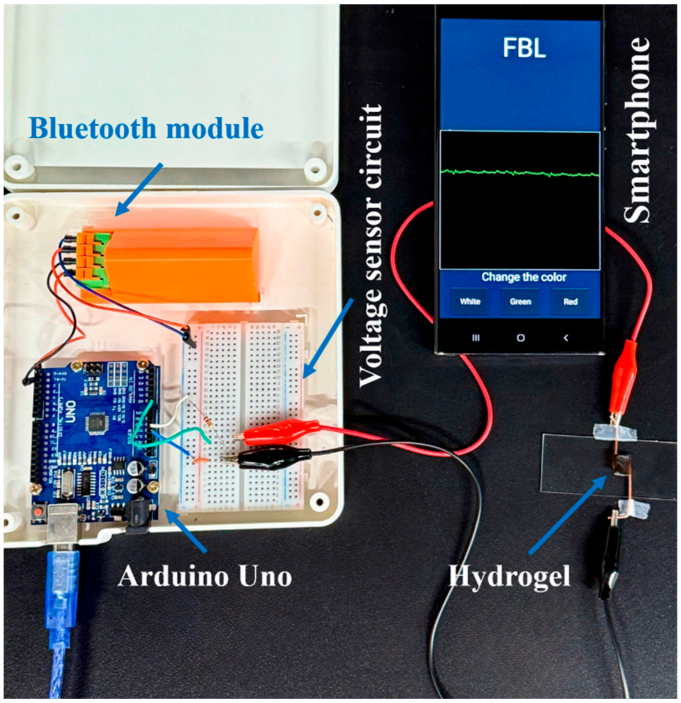


**Figure S3:** Wireless sensing communication system setup: Arduino Uno, Bluetooth module, voltage sensor circuit, and a smartphone.

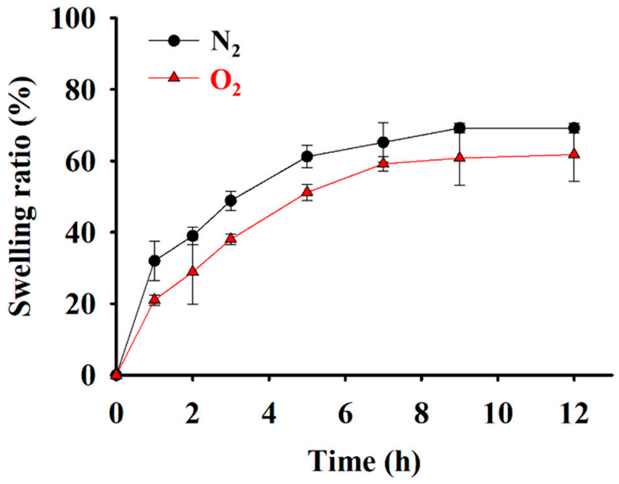


**Figure S4:** Swelling ratio profile of HDP-PVA hydrogel (100:5 wt%) in $N_2$ and $O_2$ conditions.

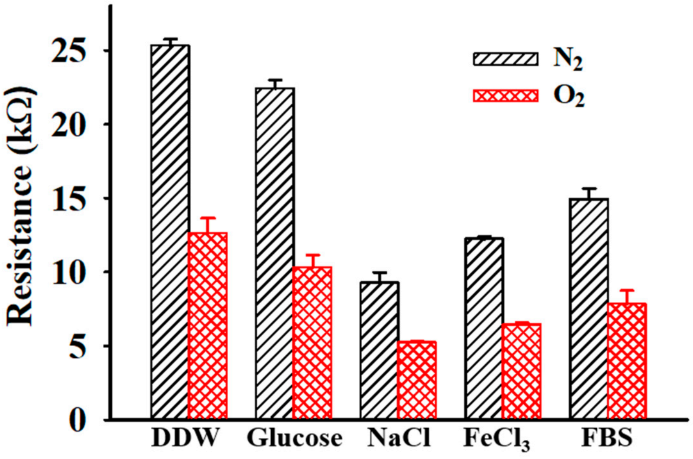


**Figure S5:** Anti-interference test of HDP-PVA hydrogel (100:5 wt%) in $N_2$ and $O_2$ conditions (n = 3).

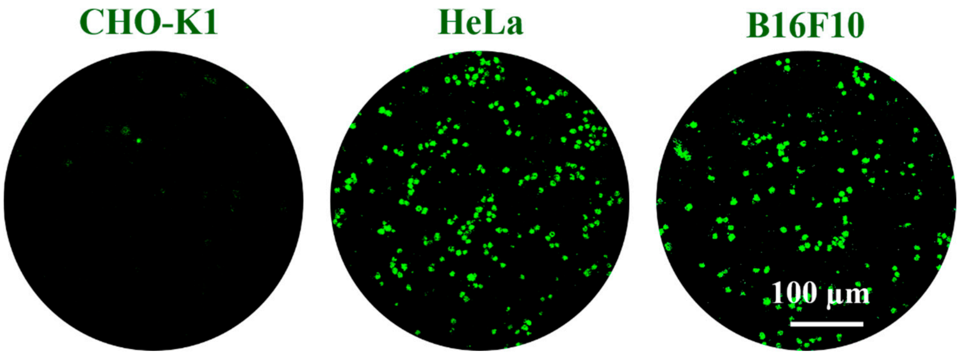


**Figure S6:** Representative images of BioTracker™ 520 Green Hypoxia Dye signal in CHO-K1, HeLa and B16F10 cells. Cell concentration $1 \times 10^5$ cells/mL.

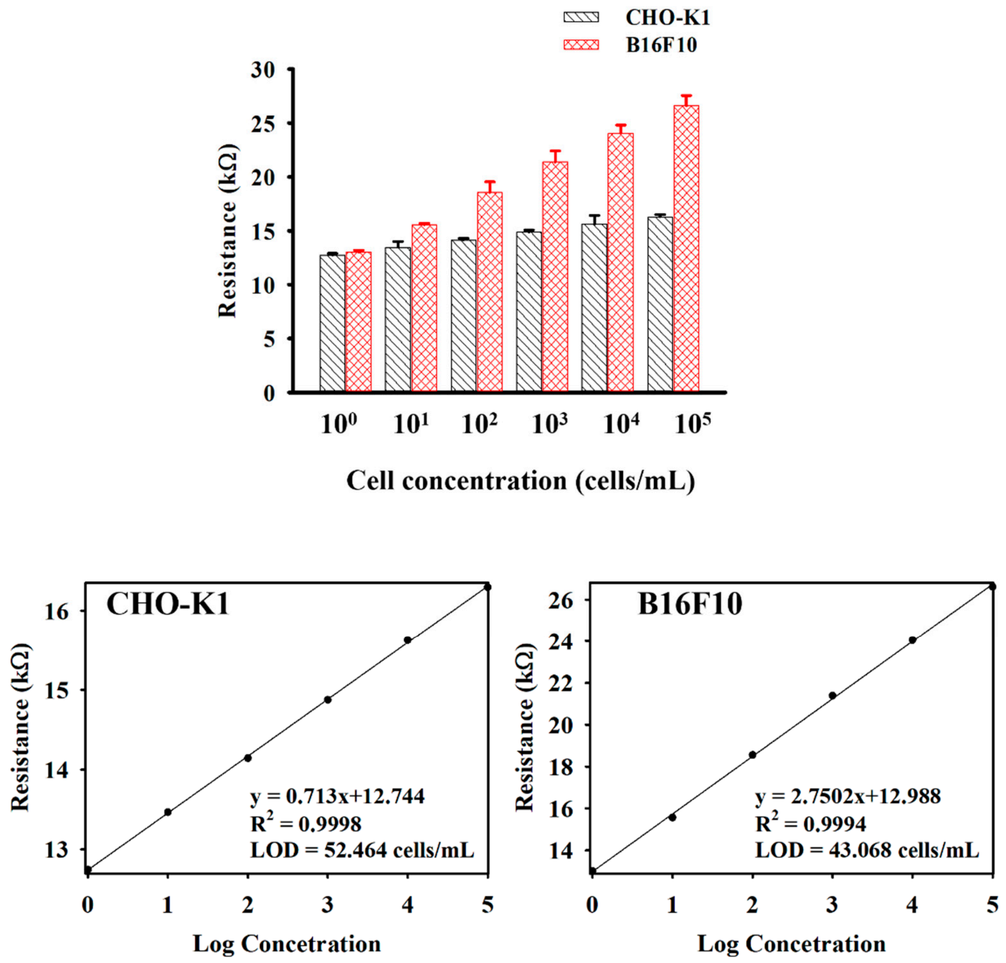


**Figure S7:** LOD data of HDP-PVA hydrogel (100:5 wt%) treated with CHO-K1 (normal cell) and B16F10 (cancer cell). Cell concentration $10^0$ - $10^5$ cells/mL.

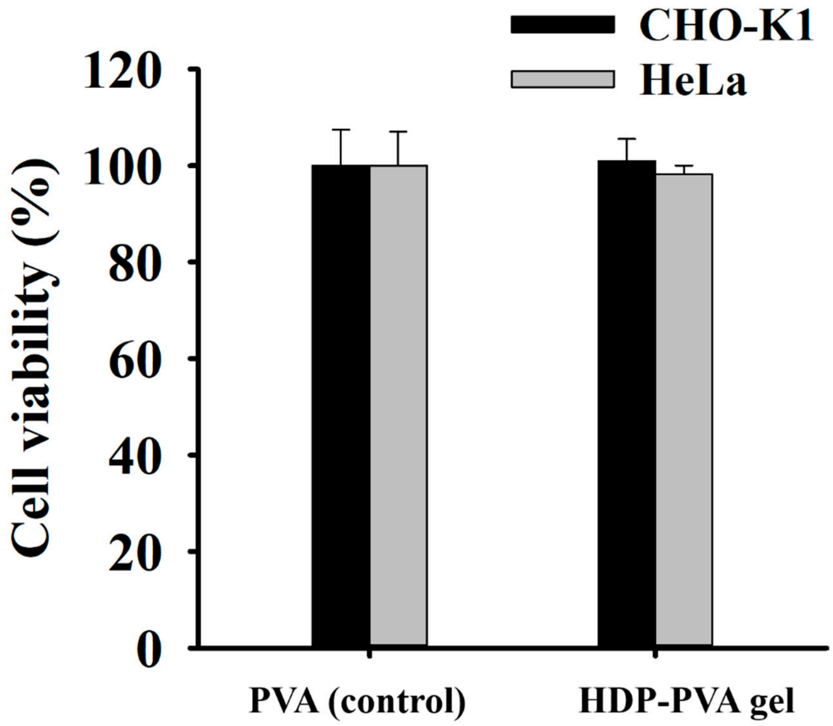


**Figure S8:** Cell viability assessment by MTT assay method using CHO-K1 (normal cell) and HeLa (cancer cell). Cell concentration $1 \times 10^5$ cells/mL.

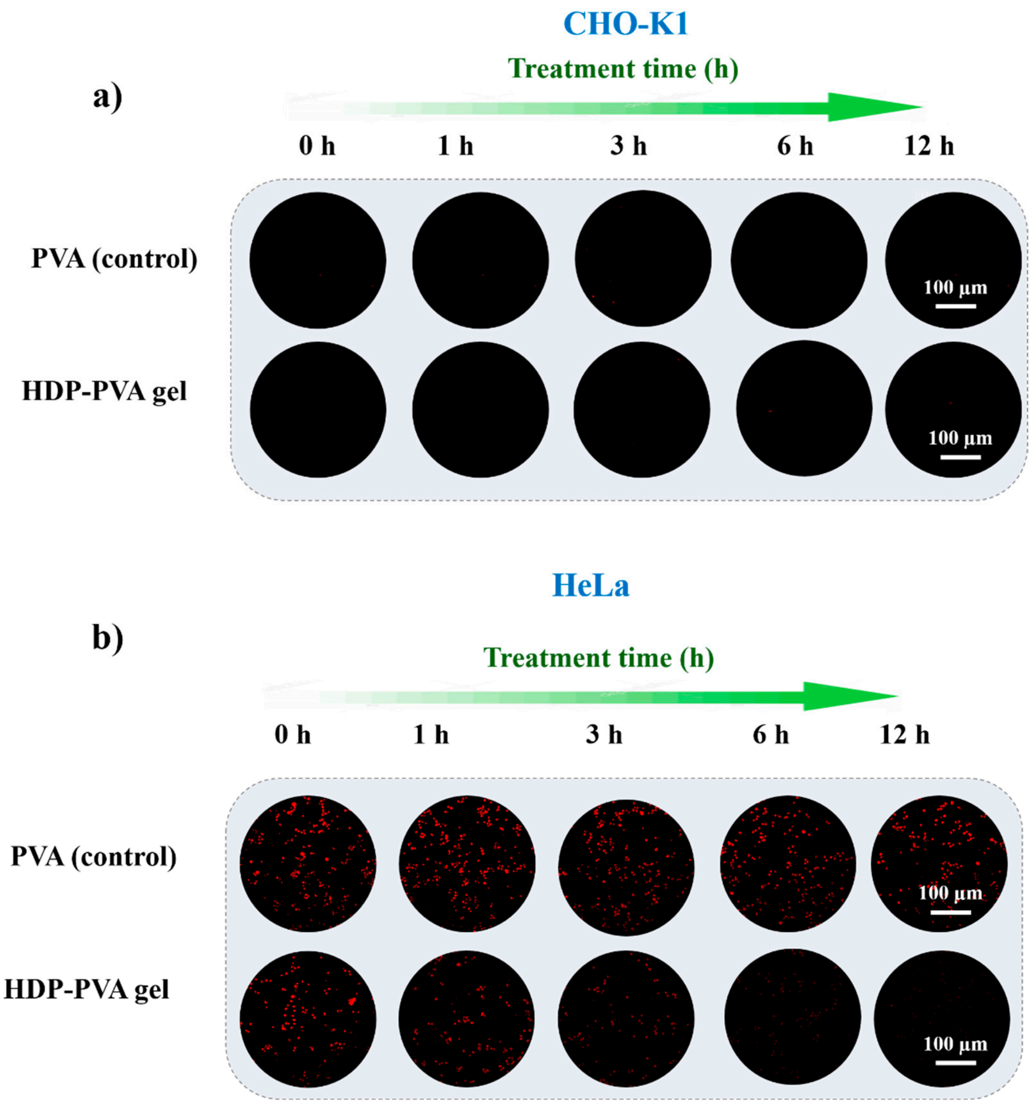


**Figure S9:** Time-dependent ROS scavenging activity of PVA and HDP-PVA hydrogel treated with **a)** CHO-K1 cells, and **b)** HeLa cells. Cell concentration $1 \times 10^5$ cells/mL.

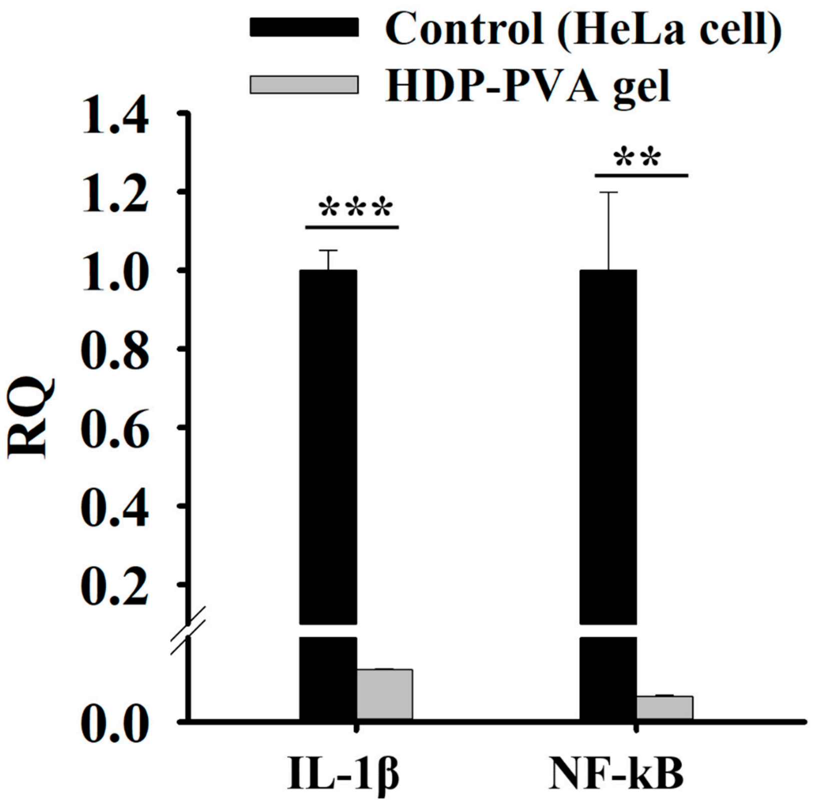


**Figure S10:** The expression levels of oncogenic signaling genes; IL-1β and NF-kB genes of HeLa cell lines after treatment with HDP-PVA hydrogel. Statistical analysis performed using Student's t-test: Paired Two Samples for Means (**$p < 0.01$ and ***$p < 0.001$ for $n = 3$ samples).
